# SARS-CoV-2 testing strategies for outbreak mitigation in vaccinated populations

**DOI:** 10.1371/journal.pone.0271103

**Published:** 2022-07-13

**Authors:** Chirag K. Kumar, Ruchita Balasubramanian, Stefano Ongarello, Sergio Carmona, Ramanan Laxminarayan

**Affiliations:** 1 Princeton University, Princeton, NJ, United States of America; 2 Center for Disease Dynamics, Economics & Policy, New Delhi, India; 3 Foundation for Innovative New Diagnostics, Geneva, Switzerland; 4 University of Witwatersrand, Johannesburg, South Africa; Regional Medical Research Centre Bhubaneswar, INDIA

## Abstract

Although COVID-19 vaccines are globally available, waning immunity and emerging vaccine-evasive variants of concern have hindered the international response and transition to a post-pandemic era. Testing to identify and isolate infectious individuals remains the most proactive strategy for containing an ongoing COVID-19 outbreak. We developed a stochastic, compartmentalized model to simulate the impact of using Reverse Transcriptase Polymerase Chain Reaction (RT-PCR) assays, rapid antigen tests, and vaccinations on SARS-CoV-2 spread. We compare testing strategies across an example high-income country (the United States) and low- and middle-income country (India). We detail the optimal testing frequency and coverage in the US and India to mitigate an emerging outbreak even in a vaccinated population: overall, maximizing testing frequency is most important, but having high testing coverage remains necessary when there is sustained transmission. A resource-limited vaccination strategy still requires high-frequency testing to minimize subsequent outbreaks and is 16.50% more effective in reducing cases in India than the United States. Tailoring testing strategies to transmission settings can help effectively reduce disease burden more than if a uniform approach were employed without regard to epidemiological variability across locations.

## 1. Introduction

Despite the growing availability of vaccines that confer immune protection against severe acute respiratory syndrome coronavirus 2 (SARS-CoV-2), cases of the 2019 coronavirus disease (COVID-19) have continued to rise in many countries [1]. The emergence of highly contagious SARS-CoV-2 variants of concern [2] that are only partially neutralized by existing antibodies or prior vaccination has resulted in widespread COVID-19 outbreaks even in highly vaccinated populations [3, 4]. Some locations, particularly in low- and middle-income countries (LMICs), still lack widespread access to vaccines and continue to experience significant excess mortality [5], making them particularly vulnerable to contagious variants of concern. All these factors must be considered when designing COVID-19 mitigation strategies to counter emerging outbreaks. Consequently, even if vaccines are distributed widely, SARS-CoV-2 will likely continue to pose a public health threat for many years because of the rapid emergence of variants of concern and waning immunity. Nevertheless, guidance is lacking on specific and tailored mitigation strategies for using both vaccinations and mass testing and containment to control an outbreak in real time.

COVID-19 outbreaks can emerge rapidly because of SARS-CoV-2’s high transmission rate and the prevalence of asymptomatic carriers [6]. Although vaccination lessens disease severity, only testing can identify, track, and quickly and preemptively contain disease spread. Currently, two main diagnostic methods are widely used [7]. *Reverse Transcriptase Polymerase Chain Reaction* (RT-PCR) assays, which are considered to be the gold standard testing method, detect the presence of viral RNA in respiratory samples [8]. Although highly sensitive, test results typically require a turnaround time of two to three days during which an infected individual may continue transmitting the virus [9]. *Later flow rapid antigen tests* can be read in less than an hour, and an individual who tests positive can immediately self-isolate [10]. However, rapid antigen tests require a higher viral load for positive detection, must be tailored to the antigens on a specific variant, and may not return a positive result for infectious individuals past the early stages of their infection [11].

Testing usually only occurs in a reactionary fashion, especially after individuals are vaccinated against COVID-19. For instance, either an individual (a) exhibits symptom that may be attributed to COVID-19, (b) comes in close contact with an infected person, or (c) seeks to travel and must show proof of a negative test. Comparatively, proactive testing administered regularly within a community–such as in a college campus, business, or factory–is more likely to be effective in mitigating an outbreak as it will capture asymptomatic spreaders in real time.

Although vaccinations, RT-PCR assays, and antigen tests can all play a role in COVID-19 containment, there is lack of clarity on the optimal coverage and frequency with (a) which of these tests should be used, (b) how they may be combined to greatest effect, and (c) the impact of vaccinations on reducing the need for testing. Moreover, while COVID-19 has affected populations around the world regardless of socioeconomic status [12], there are significant differences and variability in transmission intensity by region, country, and sub-national setting [13]. Tailored testing strategies that are specific to the local transmission context may be superior to applying a one-size-fits-all method for every population. Guidance on testing strategies may differ by transmission setting (i.e., in high-income countries, such as the United States, versus low- and middle-income countries, such as India) as not only do transmission dynamics differ, but low- and middle-income countries (LMICs) now have the majority of confirmed COVID-19 cases [13] along with more barriers to adequate health care [14]. Significant effort has been directed at containment strategies across high income countries in Europe and North America [15–18], but these findings cannot be generalized to all countries worldwide because of differences in transmission intensity. Moreover, for COVID-19 to become globally endemic, it must be controlled in all countries [19], so it is critical to determine setting-specific mitigation strategies and characterize how vaccines and testing may be used in tandem to move international public health efforts towards the post-pandemic phase.

We used a stochastic, compartmentalized, agent-based model (ABM) to simulate an emerging outbreak from a novel COVID-19 variant of concern in the United States and India under different proactive testing scenarios and vaccination potentials. We evaluated various strategies for COVID-19 control in both countries to identify their efficacy and costs and determine how testing recommendations may differ by transmission setting for mitigating an outbreak even in highly vaccinated populations. Our simulation results can be directly applied to community settings, such as office buildings, factories, or campuses, that are trying to reopen safely and with minimal disease spread.

## 2. Methods

Accurately simulating SARS-CoV-2 dynamics requires a realistic modeling framework that is parameterized by real-world COVID-19 data. We developed an agent-based model (ABM) of SARS-CoV-2 spread and simulated the impact of various testing strategies across both transmission settings (the US and India) to determine the varying efficacy of the proposed interventions. ABMs present an advantage over traditional compartmentalized models because both behavior/interactions and characteristics can be programmed down to the level of the individual, ensuring a model that is descriptive and representative of the populations being modeled. ABMs also enable the natural propagation of uncertainty in the dynamic projections of the number of infected individuals that arises from uncertainty in the data underlying the model. To parameterize the model, we gathered data on COVID-19 epidemiology in the US and India from freely and publicly available data sources.

### Data on COVID-19 illness outcomes

We obtained data on COVID-19 cases and death counts from the beginning of record acquisition in the United States and India stratified by age, gender, and comorbidity: US data were obtained from the National Center for Health Statistics (of the US Centers for Disease Control and Prevention, CDC) [20], and data from India were obtained from previous studies [13]. We used these data to estimate the probability of death from COVID-19 for each category of age, gender, and comorbidities. Next, we estimated the probability and length of a hospital stay: the CDC maintains records on the probability of hospitalization and summary statistics (i.e., the 25^th^, 50^th^, and 75^th^ percentile) for time spent in the hospital due to severe COVID-19 infection [21]. Although having the underlying distribution of hospital stay duration would be ideal, based on empirical evidence that such lengths generally follow a negative binomial distribution [22], we construct negative binomial distributions with the same summary statistics to generate an estimated probability for various lengths of hospital stays. We had different hospitalization rates in the United States and India [23], but we assume that the pattern of hospital stay length is maintained between the two countries. Ultimately, using these data, we determined the expected outcome (i.e., simple recovery, recovery after hospitalization and length of corresponding hospital stay, or death after a hospitalization) of a COVID-19 infection given an age, gender, and set of comorbidities.

### Data on SARS-CoV-2 transmission

Although the above data determine the outcomes of COVID-19 for an individual after infection, this does not detail the spread of the SARS-CoV-2 spread in a population. Thus, to simulate SARS-CoV-2 transmission, we used data on the incubation period [24], transmission probability since infection [25], and inferred viral load after symptom onset [26]. These data directly determine the time-varying (i.e., daily) probability that an infected individual will transmit to their secondary contacts and whether that individual will be flagged as infectious by a test. Yet, despite extensive data along with specified uncertainty for the incubation period, transmission probability since infection, and viral load after symptom onset of SARS-CoV-2 (see S1 Table in S1 File for the parameters used in our analysis), quantifying viral load prior to symptom onset remained challenging with contradictory evidence on the peak viral load [27, 28]. Thus, we drew from previous viral kinetics models to infer the viral load prior to symptom onset; our results are likely robust to changes in viral load distribution as there is variability in our estimated viral loads by individual (S1 Fig in S1 File). Specifically, we say that viral load peaks anywhere from day 0 to day 4 after symptom onset [29]; the peak is anywhere from 5 to 11 log10 virions per mL [26], that log10 viral load increases linearly from negative infinity on the day of infection to the aforementioned peak, and that log10 viral load decays from peak to the end of the individual’s infection linearly with a slope drawn from meta-analyses [26]. See S9 Fig in S1 File for our inferred viral load distributions. We combined our estimates of the viral load over time with data on the sensitivity and specificity of RT-PCR tests and antigen tests [8, 11, 30] to estimate the probability that an individual tests positive with a given test on set day of their infection. We also estimated the reduction in transmissibility of 75% after increasing doses of a standard messenger ribonucleic acid vaccine based on real-world data of vaccine effectiveness [31, 32].

Additionally, we used data from studies that provided the contact matrices and the distribution of the number of secondary cases arising from an infected individual for each transmission setting [13, 33]. These variables were used for determining how many infections may arise from a single infected individual and the likely age of the consequently infected individuals. S1 Table in S1 File shows a complete list of parameters compiled to simulate SARS-CoV-2 transmission in our model. From these parameters, we simulated realistic disease spread in a population across settings.

### Model description

We developed a stochastic, compartmentalized, empirically-driven agent-based model (ABM) to project COVID-19 cases, hospitalizations, and deaths given a variety of testing strategies in the US and India. We adopted the following structure in our model: individuals in the population start as “susceptible” if they have not yet been infected, or “recovered” if they have previously been infected before entry into our simulation. Susceptible individuals can become “infected and not expressing symptoms” after a positive transmission event with another infected individual. Individuals can either stay as “infected and not expressing symptoms” (i.e., “asymptomatic”) for the duration of their infection or move to “infected and expressing symptoms”. Infected and symptomatic individuals may either recover or become hospitalized. Finally, hospitalized individuals may either recover or die (see S1 Table in S1 File for the probability and duration of each event).

The individual’s probability of transmitting the virus changes daily (peaking near symptom onset), as does the viral load (peaking shortly after symptom onset). We inferred the viral load as described above based on previous studies. Nevertheless, not all individuals will transmit the virus: in accordance with “superspreading” [34], we drew the number of positive transmission events for each infected individual from location-specific negative binomial distributions [13, 35], and whom they are likely to infect from the contact matrices described above. Individuals interacted homogeneously with each other following a Brownian process in an open space with size such that R_eff_ was 2.5 without any additional testing or vaccine-based mitigation.

ABMs present two benefits over traditional deterministic compartmentalized models: (i) implementing individual specificity is easier, and (ii) they are inherently stochastic and thus can provide credible ranges of the epidemic trajectory given initial conditions. Each model was run in the following way: there were 5,000 individuals with age and genders drawn from US and Indian census data, and comorbidities drawn from recorded prevalences given age and gender in 2017 [36]. Note that these parameters can be easily changed so that policymakers can determine which mitigation and testing strategies are most effective for specific communities of interest. We ran each model for 200 days (until a steady state is reached), 200 times (i.e., independent replicates), and present the 50th, 2.5th, 25th, 75th, and 97.5th percentiles (i.e., the “credible intervals”) over the 200 independent replicates as summary statistics in figures; when reporting values in the main text, we simply provide the 50th percentile and the 2.5th and 97.5th percentiles as the credible interval. We use standard methods of propagating error in computed variables to ensure meaningful uncertainty [37]. Unless otherwise mentioned, to test for statistically significant difference in medians between two distributions, we use a two-sided paired Mood’s Median test [38]. The model was developed in Python 3 [39] using the Mesa package under the Apache2 license [40], and all analyses were also done in Python 3.

## 3. Results

Before evaluating the effect of our testing scenarios, we ran our model without any mitigation (S2 Fig; S2 Table in S1 File) to validate the model’s results and ability to recreate observed COVID-19 statistics. The model-estimated case-fatality ratio (CFR) for the United States was 2.66% [95% percentile credible interval from 200 independent realizations of the ABM, 2.17–3.12%], and the actual CFR at time of writing is 3.05% [20]; likewise, the model-estimated CFR for India was 1.85% [1.47–2.25%] and the actual CFR based on available data was 2.11% [13]. Since our model recreated the overall expected dynamics, we could justify the use of this model to identify the efficacy and trade-offs of testing strategies and vaccinations in both settings.

### Proactive testing to mitigate an emerging outbreak

**Fig 1:**
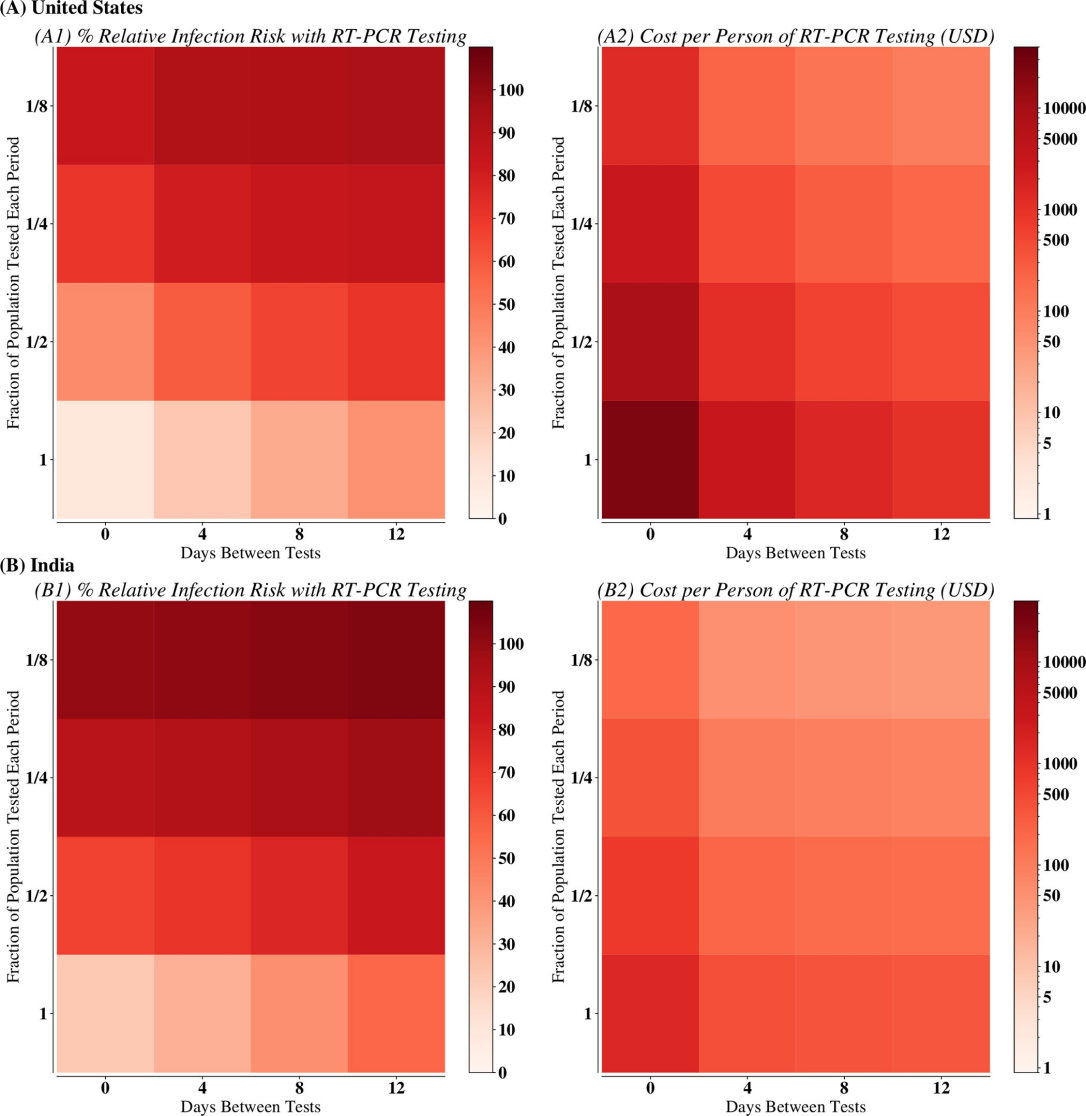
Relative infection risk and cost of testing for various RT-PCR scenarios in (A) United States and (B) India. Subplot (1) shows the relative infection risk of RT-PCR tests being used at the given frequency and coverage, compared with the scenario with no mitigation. Subplot (2) provides the cost of that testing scenario assuming that all susceptible individuals are tested at the given frequency and coverage.

**Fig 2:**
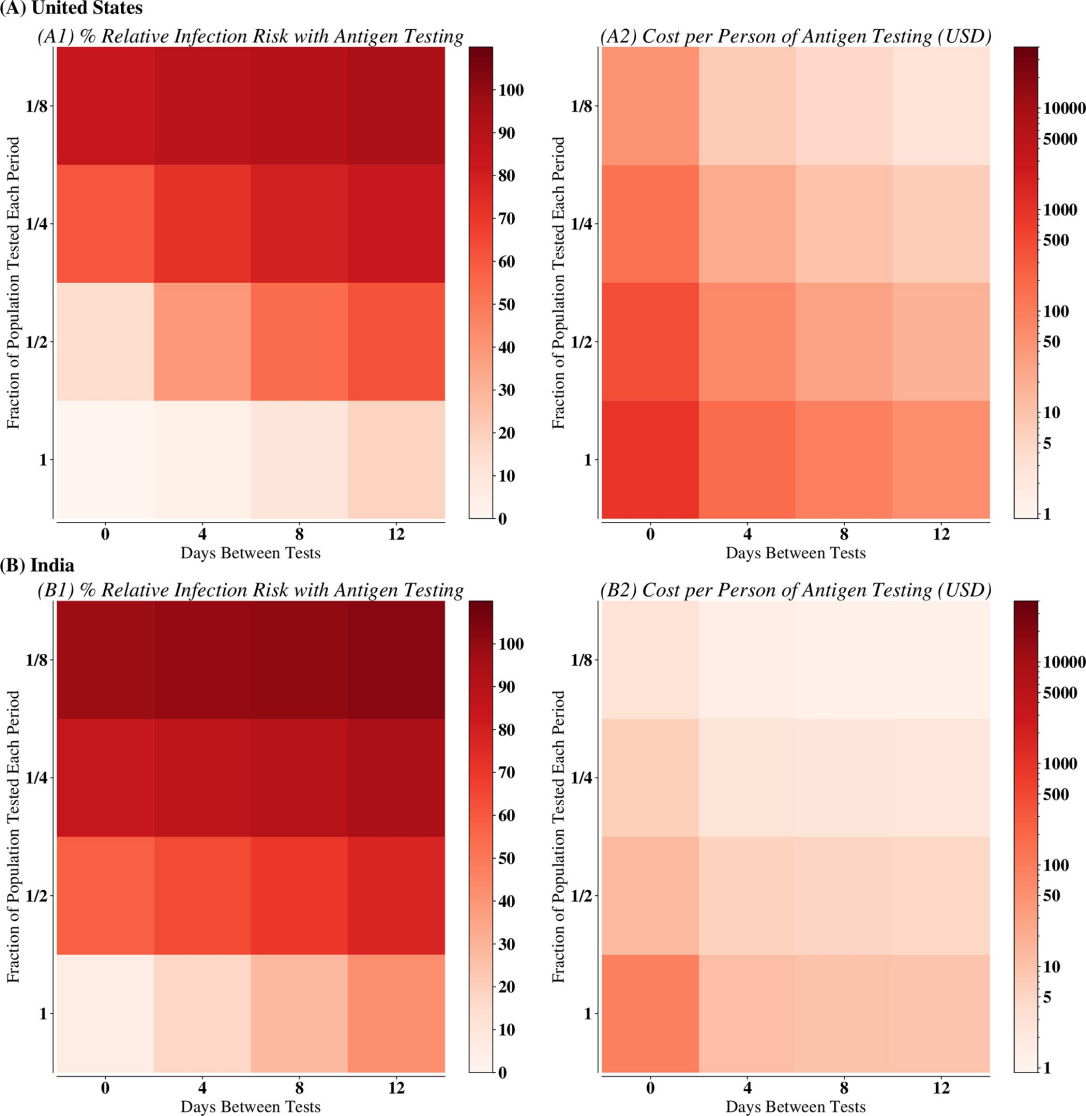
Relative infection risk and cost of testing for various antigen scenarios in (A) United States and (B) India. Subplot (1) shows the relative infection risk of antigen tests being used at the given frequency and coverage, compared with the scenario with no mitigation. Subplot (2) provides the cost of that testing scenario assuming that all susceptible individuals are tested at the given frequency and coverage.

The B.1.1.529 SARS-CoV-2 variant (designated “Omicron” by the World Health Organization) is almost completely evasive to existing vaccines or immunity from prior infection [41]. Based on Omicron’s observed infectivity even in highly vaccinated populations, we modeled the impact of RT-PCR (Fig 1) or antigen testing (Fig 2) on a representative population in the US or India by proactively testing at various frequencies (days between subsequent proactive administration of testing) and coverages (fraction of people tested each time proactive screening is done). While the cost of the surveillance depended only on the rate of testing (i.e., the average number of tests administered per day), the risk of infection depended on the frequency, coverage, type of test used, and transmission setting, with the risk of infection from each test and setting responding differently to changing frequency and coverage (finer granularity of varying frequency and coverage shown in S3-S5 Figs in S1 File).

Overall, for the same coverage and frequency of testing, using RT-PCR assays resulted in 12.65% [7.01%– 18.30%] more cases than antigen tests in the US and 9.30% [4.75%– 13.85%] more cases in India (Figs 1 and 2), likely because RT-PCR tests require a two-to-three-day turnaround time during which infected individuals may continue transmitting (S6 Fig in S1 File). Rapid antigen tests are also up to 20 times cheaper, allowing for further screening than RT-PCR assays with the same financial budget (Fig 2). Ultimately, when used frequently and widely, antigen tests were notably better than RT-PCR assays (S3-S5 Figs in S1 File). Nevertheless, at low coverage and frequency, neither test was effective at mitigation.

As a potential tool for policymakers to determine the impact of various testing strategies without needing to run complete simulations, we developed explanatory regression models that predicted the percent of individuals infected by setting from the frequency, coverage, and type of test used (*R*^*2*^ of all models >90%; S3-S5 Tables in S1 File). These models indicated that on average maximizing frequency had a larger effect on mitigating cases than maximizing coverage independent of the test type used. Specifically, given a financial constraint on the amount of testing available, the models suggested that the frequency of testing should be 3.71 [2.99–4.43] and 5.01 [4.15–5.87] times greater than the coverage of testing in the United States and India, respectively, to optimally reduce cases the most.

While we observe the same general trends in both settings, increasing frequency is more important for (a) antigen testing and (b) in India: Figs 1 and 2 show a strong gradient of increasing cases as testing frequency is decreased, most noticeably for antigen testing and in all testing strategies in India. First, the two types of tests have different factors that dominate their efficacy. There is a greater change in the infection risk when increasing frequency when using antigen testing rather than RT-PCR assays. Comparatively, there is a greater impact of increasing coverage when using RT-PCR assays. Secondly, the two settings have different factors that determine testing strategy efficacy. While high-frequency, low-coverage testing can still be useful in the United States, coverage in India must be high for effective mitigation. Although increasing coverage when it was low had little benefit in both transmission settings, increasing coverage from half of the population surveilled to the whole population surveilled had a larger effect in India (Figs 1 and 2). Ultimately, while increasing frequency was the overall driver for reducing cases, in certain testing scenarios in India, our simulations suggest that increasing coverage remains critical. Finally, we conducted sensitivity analyses by running example strategies with R_eff_ from 2.5 (used in all main figures and analyses;S7, S8 Figs in S1 File) and decreasing R_eff_ from 2.5 to R_eff_ = 2 (S9 Fig in S1 File) and 1.5 (S10 Fig in S1 File). Our main findings and trends still held across all values of R_eff_, though there are fewer cases with a lower R_eff_.

### Utilizing combinations of tests and vaccines

**Fig 3:**
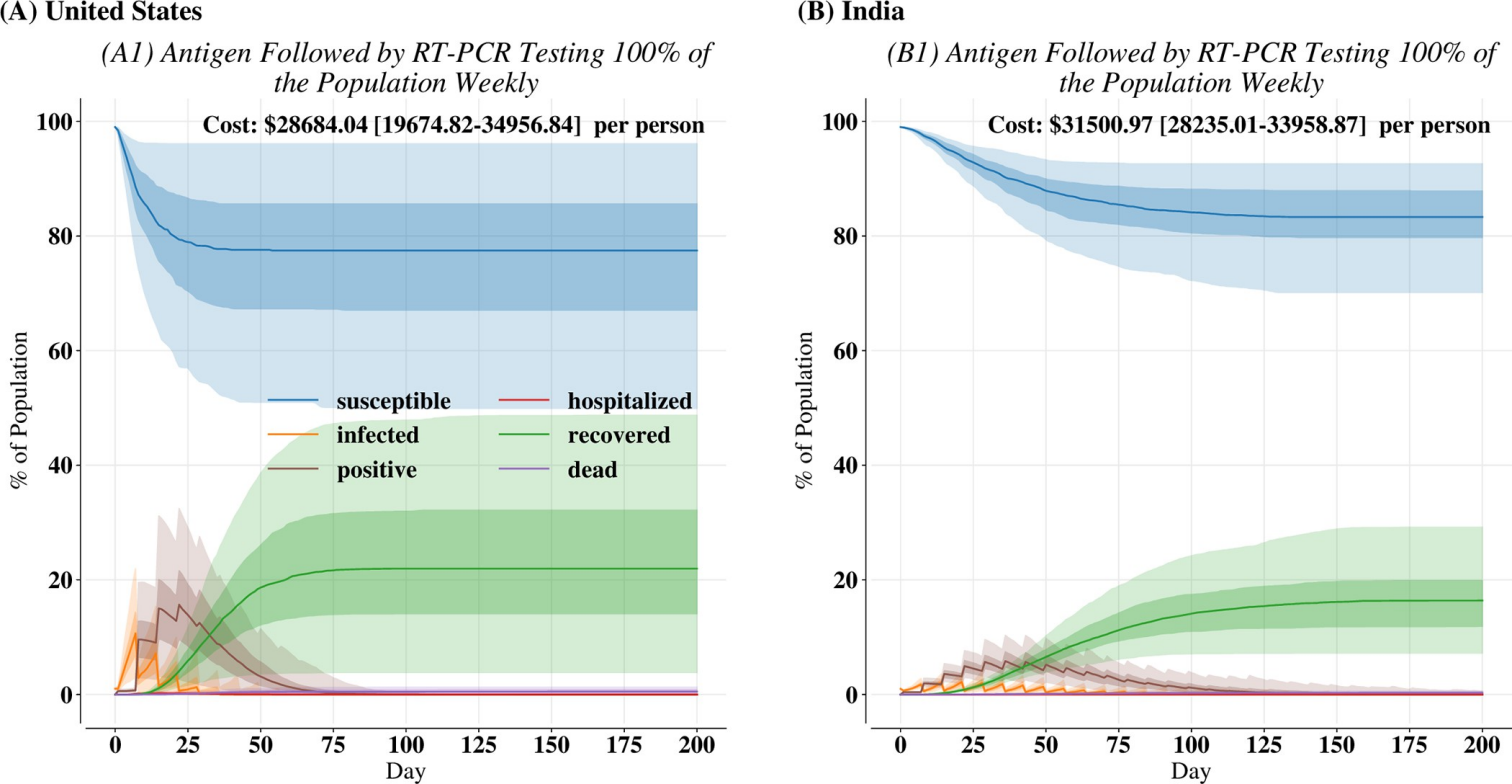
Disease course in (A) United States and (B) India for antigen testing followed by RT-PCR testing of antigen-negative individuals 100% of the population weekly. Bold lines are the median over 200 independent replicates. Dark-shaded regions are the 25^th^ to 75^th^ percentiles over the 200 independent replicates while light-shaded regions are 2.5^th^ to 97.5^th^ percentiles.

Our simulations suggested that antigen tests and RT-PCR assays excel in complementary regimes: antigen tests were most effective when used frequently whereas RT-PCR assays were more effective when used widely. Thus, we proposed mixed strategies that combined the different types of tests along with vaccinations to best address an emerging outbreak driven by a variant of concern that may be more transmissive and vaccine-evasive. To identify and isolate infectious individuals as quickly as possible, we proposed testing all individuals weekly with antigen tests and following up all negative results with an RT-PCR assay (Fig 3). Since RT-PCR assays have higher sensitivity, their use as a follow-up should allow for the detection of individuals with a viral load too low to be detected by an antigen test. Nevertheless, this strategy was resource intensive: it potentially required more than just one test per individual.

Such a mixed strategy was effective in both settings—resulting in minimal hospitalizations and deaths—but more effective in reducing cases India than in the United States. Compared with weekly antigen testing (S8 Fig in S1 File), this approach did not significantly reduce cases in the United States (*λ* *=* 2.25, *p =* 0.13) or India (*λ* = 1.00, *p* = 0.32). Nevertheless, this strategy resulted in reaching 1% incidence 7.0 [0.0–14.0] (*λ* *=* 9.25, *p* < 0.005) days earlier in the United States and 7.0 [–7.0–7.0] days earlier (*λ* *= 5*.*81*, *p* = 0.016) in India. When faced with an outbreak from a contagious variant of concern, this simulation shows that effective surveillance is critical in the early stages of transmission to mitigate disease burden and supplementing antigen testing with RT-PCR testing allows for a faster reduction in disease burden.

**Fig 4:**
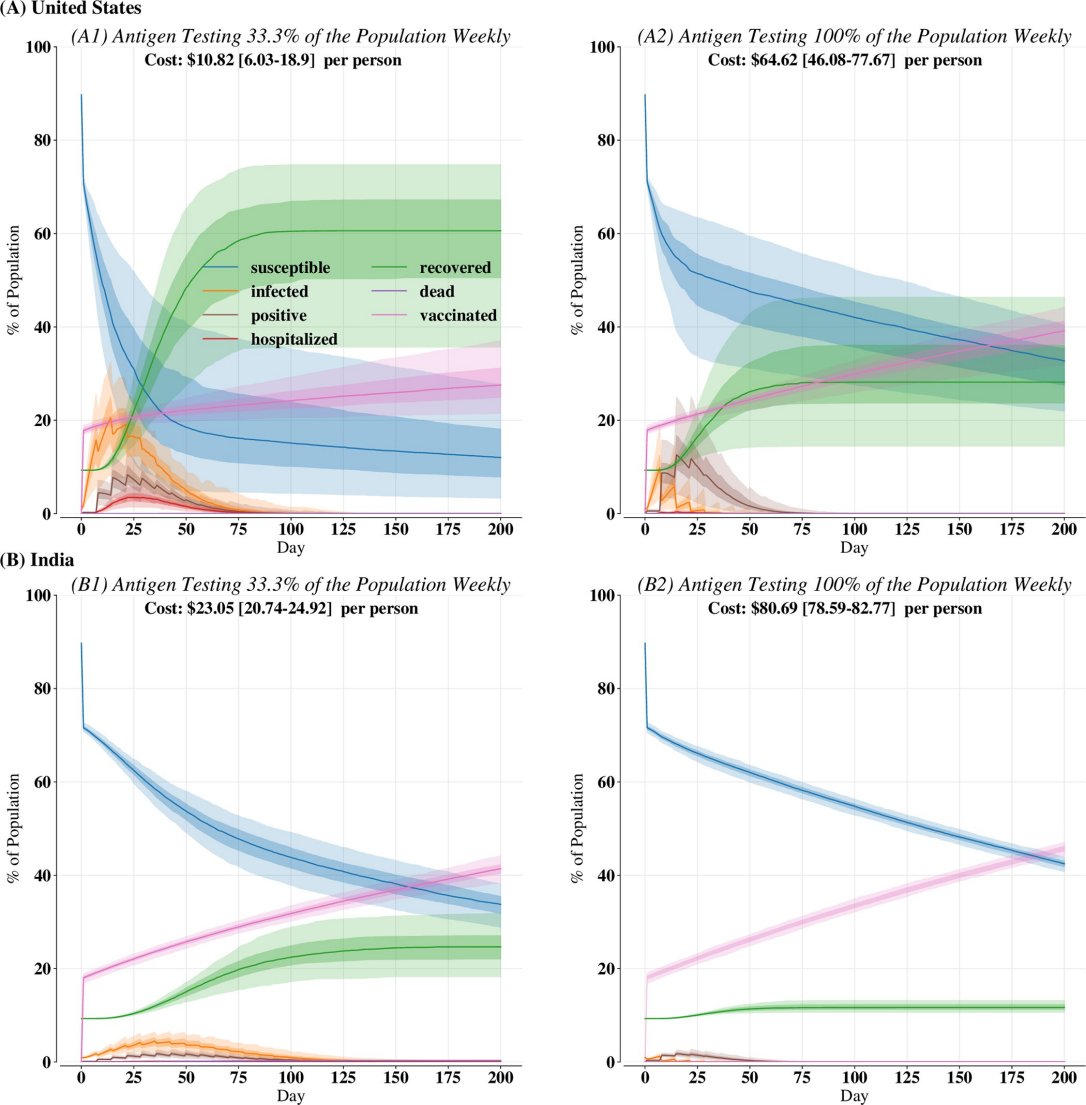
Disease course for (A) United States and (B) India with 20% of the population as initially vaccinated and then 0.25% of the susceptible population being vaccinated each subsequent day; vaccines are coupled with antigen testing (1) 33.3% or (2) 100% of the population weekly. Bold lines are the median over 200 independent replicates. Dark-shaded regions are the 25th to 75th percentiles over the 200 independent replicates; light-shaded regions are 2.5th to 97.5th percentiles.

Likewise, the rapid emergence of variants of concern has placed an emphasis on widespread vaccination campaigns and even booster shots for some populations. Nevertheless, key questions remain about optimal testing strategies and epidemic trajectories for populations as vaccines are administered or additional immunity is conferred through booster shots. Thus, we determined how proactive testing and simple vaccination strategies could be used together to promote SARS-CoV-2 endemicity. We coupled an increasing fraction of the population becoming increasingly vaccinated (i.e., receiving a first vaccine dose if unvaccinated or a second or third dose if eligible) with antigen testing 100% of the population weekly and antigen testing 33.3% of the population weekly in both settings (Fig 4; see S8 Fig in S1 File for these scenarios but without vaccines).

Overall, vaccines are mostly effective in reducing COVID-19 cases at both coverages and in both transmission settings. First, compared to there being no vaccinations or immunity, 20% of the population being initially completely vaccinated (i.e., having received all available vaccine doses and not yet suffering from waning immunity) coupled with weekly antigen testing 100% of the population resulted in 15.12% [6.39–25.50] (*λ* *= 396*.*01*, *p* < 10^−6^) fewer cases in India than without vaccines. However, the limited distributions of vaccines only reduced cases marginally in the US compared to weekly antigen testing alone (Fig 4) (*λ* *= 1*.*69*, *p* = 0.19). Secondly, we observe that the increasing number of vaccinations resulted in a mitigated disease course; concretely, we observed that vaccines coupled with antigen testing 100% of the population weekly resulted in infections below 0.5% incidence 7.0 [0–15.0] (*λ* *= 45*.*00*, *p* < 10^−6^) days faster in the US and 70.0 [34.83–126.0] (*λ* *= 372*.*49*, *p* < 10^−6^) days faster in India than the same testing strategy but with no vaccines. However, we also see that widespread testing is still critical, especially in early phases of vaccine distribution where there are many antigen-positive SARS-CoV-2 cases being captured. We found marked differences between testing 100% of population weekly or 33.3% of the population weekly: specifically, testing 100% of the population weekly resulted in 30.83% [20.41–33.10] (*λ* *= 342*.*25*, *p* < 10^−6^) fewer cases in the US and 12.99% [7.65–18.58] (*λ* *= 396*.*01*, *p* < 10^−6^) fewer cases in India than testing 33.3% of the population weekly, suggesting high frequency testing remains critical even with vaccines.

Nevertheless, while vaccines are overall effective, there are key differences in the disease course and number of infections by transmission setting. Cases peak earlier in the US than in India, so by the time more of the population has been vaccinated, there have already been more cases in the US. Specifically, there were 16.50% [3.83–33.17] (*λ* *= 396*.*01*, *p* < 10^−6^) fewer cases in India than the US when antigen testing 100% of the population weekly. Consequently, more of the population remains susceptible in India. To make our results useful for informing policy, our simulations account for varying vaccination rates and priority among those who have not yet contracted COVID-19 versus those who have. Because we project that there are more susceptible individuals in India after vaccinating, we anticipate a greater need for vaccinations to minimize a future outbreak due to an emerging variant of concern.

## 4. Discussion

### Maximizing the effectiveness of testing strategies

Though COVID-19 vaccines and tests are become widely accessible, there is a lack of insight on how to best use these methods to mitigate an emerging outbreak due to a novel and contagious variant of concern. Using a simulation-based approach that is data-informed and considers differences across transmission settings, our results provide insight into constructing tailored testing and vaccination strategies with maximum effectiveness. First, on average, antigen tests are more effective than RT-PCR tests across both transmission settings because they enable faster action to reduce transmission even though they are overall less sensitive; our results agree with real-world evidence that antigen tests have been used successfully in nation-wide testing campaigns [42, 43]. The increased mitigation of antigen tests compared with RT-PCR assays with standard turnaround times is most pronounced when 100% of the population is tested weekly. Nevertheless, screening with RT-PCR assays becomes comparable to screening with antigen tests if RT-PCR turnaround time is decreased [44] (S2 Fig in S1 File), and screening with RT-PCR assays is more effective if frequency cannot be increased but coverage remains high.

Using antigen tests results in a lower peak of daily cases compared with RT-PCR assays. Because disease spread is greatest in the early stages, when most of the population is still susceptible, early isolation of infected individuals is critical to mitigating disease spread, especially when considering highly contagious and vaccine-evasive variants of concern. Additionally, SARS-CoV-2 transmission to secondary individuals is significant even soon after initial infection, further underscoring the need for isolating infectious individuals quickly.

We show that that high-frequency testing must be prioritized when fighting an emerging outbreak driven by a contagious variant of concern, similar to the Omicron variant or other novel variants, though the relative importance of frequency versus coverage differs by setting and the type of test used. Maximizing frequency has the greatest importance for antigen testing. This is likely driven by its lower sensitivity but quicker turnaround: since antigen tests are unable to detect infected individuals with low viral loads, they must be used frequently to identify when individuals become infectious past detectable levels and force them to isolation. Moreover, frequent use of antigen tests is not substantially better than even more extensive disease mitigation strategies, such as coupling antigen and RT-PCR tests. On the other hand, RT-PCR assays can still be effective when used widely because they are more likely to identify infectious individuals with low viral loads.

### Comparison of testing effectiveness between the United States and India

Although we observe mostly similar trends in mitigation strategies between the two countries, some differences are important for tailoring mitigation solutions. First, while both transmission settings have the same R_eff_ and are parametrized with the same incubation period and transmission distribution, the disease trajectory is markedly different. Overall, transmission is shorter, reaches a higher peak in percentage infected, peaks earlier, and the credible intervals are substantially larger in the United States than India—which can be related to the contact matrices and the inherent variability in contact patterns [13]; moreover, our findings generally agree with real world analyses of SARS-CoV-2 spread in the US [45, 46]. Notably, the large credible intervals in the United States are likely driven by the high variability in the distribution of infected secondary contacts [35].

While our simulations overall indicate that high-frequency testing must be an urgent priority overall, we also find that the importance of frequency and coverage differs by transmission setting. Whereas increasing frequency is overall more important in India, increasing coverage beyond half of the population surveilled each time testing is made available substantially improves containment efforts and makes the benefits of further increasing frequency most noticeable. Ultimately, the need for widespread and frequent antigen testing is urgent in both countries, but the trade-off between frequency and coverage should be tailored to community needs.

Moreover, we find antigen testing is not only more effective but also substantially cheaper than use of RT-PCR assays. Our simulations show that given a constant budget constraint, antigen testing can be done more frequently or at wider coverage and result in fewer cases than use of RT-PCR assays. Nevertheless, we also observe that the same testing scenario may have different costs in the United States versus India. In our simulation, we assume that all individuals who have not been infected must be tested. Since in the United States the peak in cases occurs earlier, more individuals are infected in the early stages and thus a typical individual is removed from the testing pool faster than in India. Although the cost of testing thus should be lower in the United States and we do observe this in many of our simulations, in certain scenarios (e.g., where antigen tests are used at high frequency and coverage; Fig 2), the cost is less in India because the testing strategy is less effective. Since in India more individuals are infected and do not need to be tested, the cost of the strategy falls.

However, given the differential nature of disease spread, testing frequency and coverage can change as the epidemic progresses, which may also change the cost (not considered in our simulations but a viable area of future work). We also do not consider the cost of hospital beds or self-isolation, which likely differ between settings. We also do not consider factors such as human genetics that may impact the spread of a virus and viral transmission across populations. Additionally, our analysis does not explicitly consider contact tracing or self-isolation of individuals who experience symptoms, so our results more directly indicate the impact of proactive testing and immediate quarantining. Moreover, while our findings are broadly applicable to other high-income countries besides the US because of the similarities in the impact of COVID-19 across these nations, it is more challenging to confidently expand our insights to other LMICs than India because of limited data on COVID-19 in such nations. Nonetheless, our modeling agrees with the efficacy of antigen testing in many LMICs, and most importantly our results overall indicate that COVID-19 testing strategies must be tailored to the transmission setting and remain useful when considering variants of concern. Ultimately, the modeling approach in this paper presents a useful tool for policymakers and decision makers in determining the testing strategies that are likely to be effective in a specific location and how those strategies can be combined with increasing vaccinations to contain an emerging outbreak.

Finally, vaccines and testing can be combined to create containment strategies that mitigate the duration of sustained transmission and can usher in an endemic phase earlier. Our modeling recreates real-world observations of the impacts of community-level immunity due to increasing vaccinations and that cases subsequently decrease [47] due to “herd immunity”. As such, we can only provide comparative analyses of the impacts of various testing strategies when coupled with vaccines because it is challenging to discern the impact of vaccinations and subsequent herd immunity versus testing in reducing COVID-19 cases. Nevertheless, even a resource-limited vaccine allocation strategy of simply distributing vaccines randomly to susceptible individuals in addition to testing some of the population weekly is effective in minimizing cases and ending sustained transmission earlier (Fig 4). However, increasing vaccinations has different impacts in each transmission setting. In particular, the random allocation vaccination strategy utilized here is more effective in reducing cases in India than the US. This is likely due to the sustained nature of SARS-CoV-2 transmission observed naturally in India (S2 Fig in S1 File); consequently, the impact of continued vaccinations is greater in India as supposed to the US where infections peak much earlier. Widespread testing remains critical, especially in the early phases of vaccine distribution when vaccines are limited. Moreover, testing will likely continue to be necessary as further variants of concern that may be vaccine-resilient or even vaccine-resistant continue to emerge and must be monitored to ensure that resurgences of SARS-CoV-2 infections do not occur.

Generally, the most effective of the testing strategies discussed in this paper are frequently not the most expensive but rather those that are most closely tailored to the dynamics of the setting. Therefore, identifying transmission dynamics across a wide range of settings and applying specialized testing scenarios to specific environments are critical to effective mitigation. Our study suggests that contact matrices specific to the setting must be used as opposed to generic contact matrices commonly used in modeling studies [48]. Our simulation shows that social mixing patterns affect the efficacy of mitigation strategies. However, we acknowledge that in developing tailored scenarios, considering social factors is also crucial, since health behaviors, such as weight or preventative care for protecting against disease transmission, have been shown to be related to social clustering [49–51]. Nevertheless, we believe the trade-offs presented in this paper present a useful set of heuristics that can inform testing strategies and health policy across a wide range of settings.

## Supporting information

S1 File Supplementary figures and tables referenced in the main text. (PDF)
