# Supplementary material for: SARS-CoV-2 testing strategies for outbreak mitigation in vaccinated populations
Source: PLoS One. 2022 Jul 13;17(7):e0271103. doi: 10.1371/journal.pone.0271103 (PMC9278727; doi:10.1371/journal.pone.0271103)
Supplement: S1 File — (PDF) [file pone.0271103.s001.pdf]

SARS-CoV-2 testing strategies for outbreak mitigation in vaccinated populations

Supplemental Materials

7

Table S1. Model Parameters

Parameter	Value	Reference	Notes
R_0	2.5	US CDC National Center for Health Statistics	US CDC's best guess
Incubation period	Lognormal with μ = 1.63, $\sigma = 0.5$	McAloon et al. (2020)	From meta-analysis
Duration of infection given not hospitalized and recovered	Normal distribution with μ = 20.5, σ = 6.7	Hoertel et al. (2020)	Used in many modelling studies
Duration of infection prior to hospitalization: median (IQR)	18-49 years: 6 (3, 10)<br><br>50-64 years: 6 (2, 10)<br><br>≥65 years: 4 (1, 9)	US CDC National Center for Health Statistics	Estimates may change as trends change
Duration of infection given death: median (IQR)	18-49 years: 15 (9, 25)<br><br>50-64 years: 17 (10, 26)<br><br>≥65 years: 13 (8, 21)	US CDC National Center for Health Statistics	Estimates may change as trends change
Probability of admission to ICU given hospitalization: median (IQR)	18-49 years: 23.8%<br><br>50-64 years: 36.1%<br><br>≥65 years: 35.3%	US CDC National Center for Health Statistics	Estimates may change as trends change
Time spent in hospital: regular bed: median (IQR)	18-49 years: 3 (2, 5)<br><br>50-64 years: 4 (2, 7)<br><br>≥65 years: 6 (3, 10)	US CDC National Center for Health Statistics	Estimates may change as trends change
Time spent in hospital: ICU: median (IQR)	18-49 years: 11 (6, 20)<br><br>50-64 years: 14 (8, 25)<br><br>≥65 years: 12 (6, 20)	US CDC National Center for Health Statistics	Estimates may change as trends change

Table S1 (cont.). Model Parameters

Parameter	Value	Reference	Notes
Infectiousness over time	Shifted gamma distribution starting at -12.27, shape = 20.516, rate = 1.592	He et al. (2020); corrected by Slifka et al. (2020)	The papers also provide uncertainties around these parameter estimates; we draw the parameters from the uncertainty distribution to construct the final gamma distribution.
Dispersion parameter for number of contacts infected	India: 0.51; US: 0.16	Laxminarayan et al. (2020); Endo et al. (2020)	
Testing sensitivities	RT-PCR: 100% viral load>0.91 log10 copies/mL<br><br>Antigen: 100% for viral load > 2 log10 copies/mL; 60% for viral load < 2 & >1 log10 copies/mL; 33.3% for viral load < 1 log10 copies/mL & > 0 log10 copies/mL; 26% for viral load < 0 log10 copies/mL<br><br>Antibody: 96%	Kleiboecker et al. (2020); Hirotsu et al. (2020); Stites and Wilen (2020)	
Test specificities	RT-PCR: 95%<br><br>Antigen: 100%<br><br>Antibody: 95%	Stites and Wilen (2020); Hirotsu et al. (2020); Zhang et al. (2020)	
Test costs	Antigen & antibody: $5, RT-PCR: $175	Krouse and Abbot (2020)	

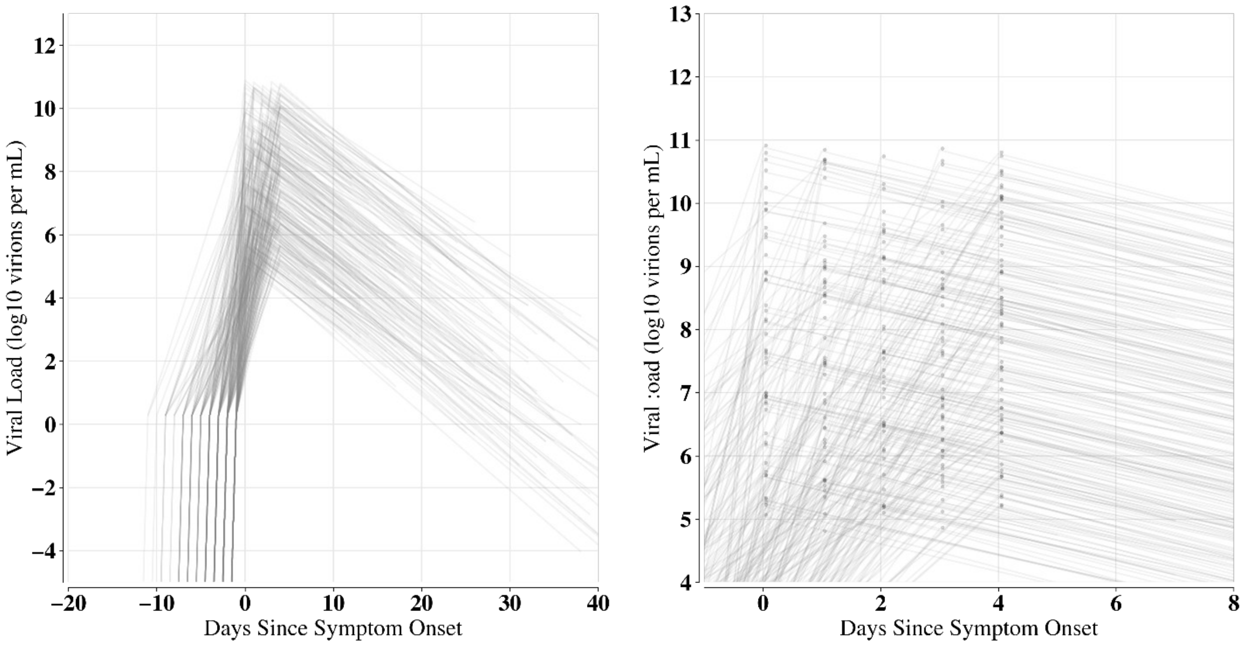

(A) United States

(A1) Summarized Disease Course *(A2) Individual Runs of Disease Course*

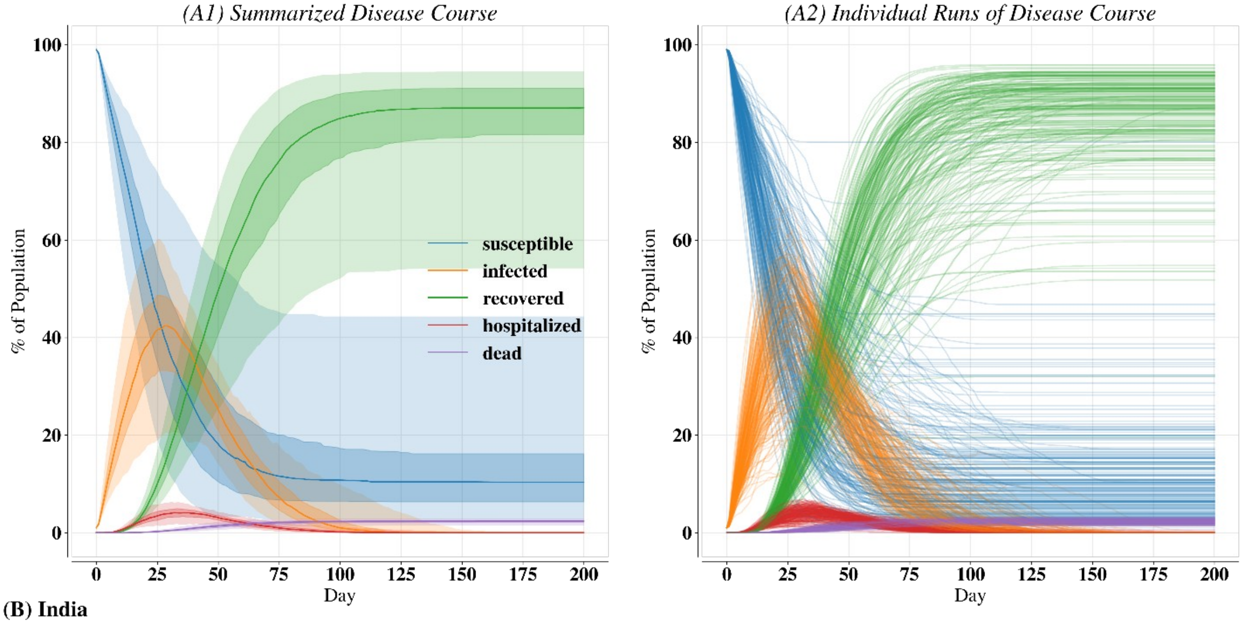

(B) India

[illegible]

Figure S1: Viral load from a simple viral load kinetics model described in the Methods section. (Left) viral load over all days and (right) viral load zoomed in on days zero to four with dots on the peak viral load. Viral load increases linearly from negative infinity log10 virions per mL to a max from 5 to 11 log10 virions per mL that occurs on days zero through four. Log10 viral load then decreases linearly to the end of infection.

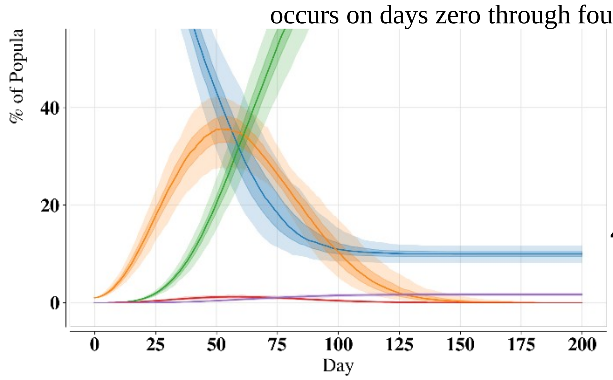

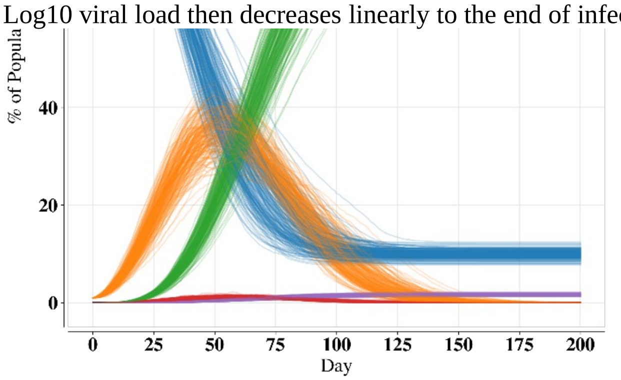

model] for the exploratory testing scenarios in Fig. 1.

	RT-PCR		Antigen	
% of population tested weekly	33.3	100	33.3	100
% infected in US	84.22 [34.164 – 93.234]	71.19 [29.018 – 87.774]	67.65 [19.389 – 88.388]	20.09 [4.820 – 48.936]
% infected in India	85.52 [82.916 – 87.681]	74.28 [65.596 – 78.883]	80.75 [75.623 – 83.562]	17.50 [7.733 – 29.502]

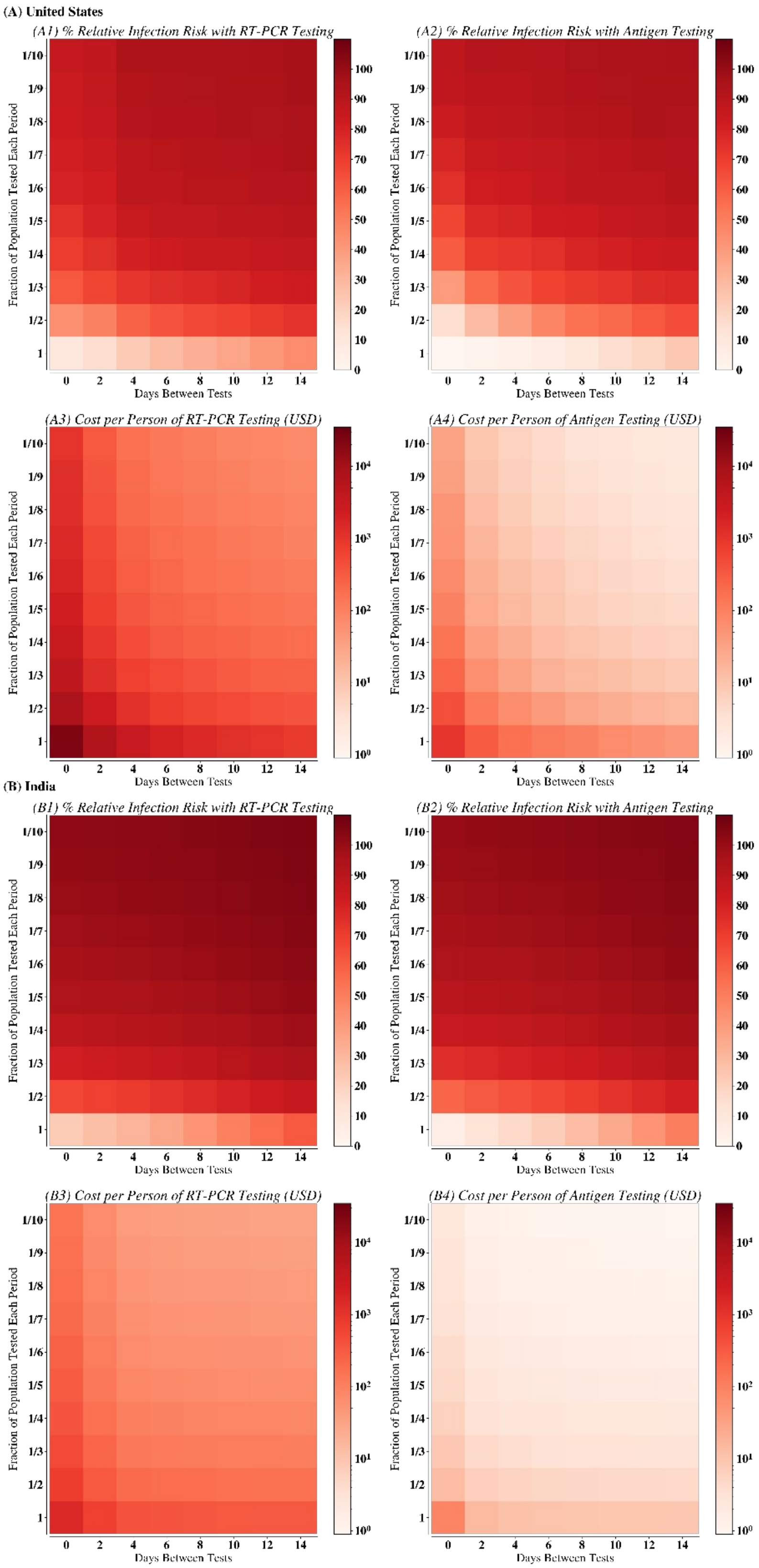

Figure S3: Complete values for limited data shown in Figs. 1 and 2.

19

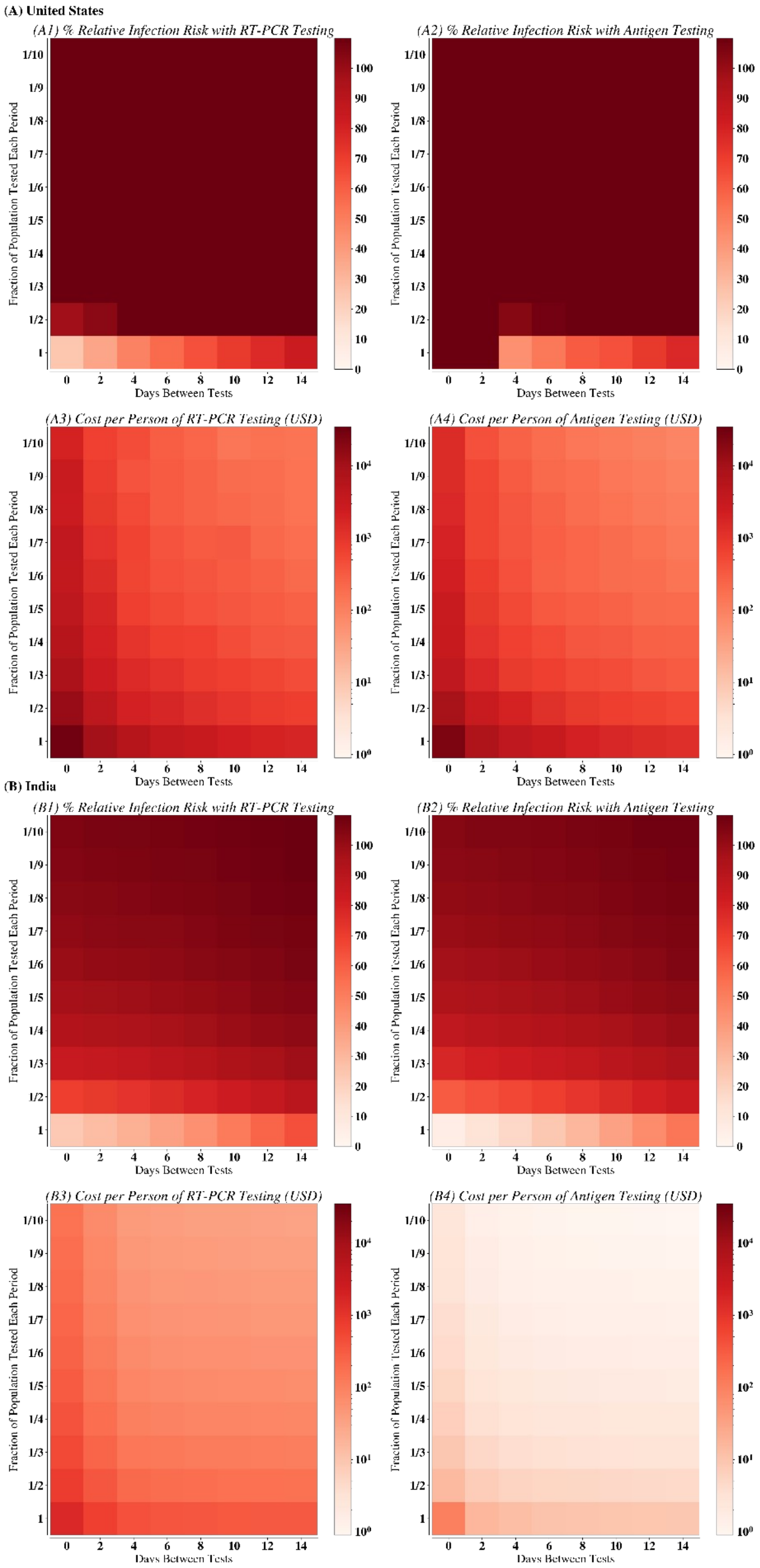

Figure S4: Upper bound of results shown in Fig. S3 and Figs. 1 and 2.

20

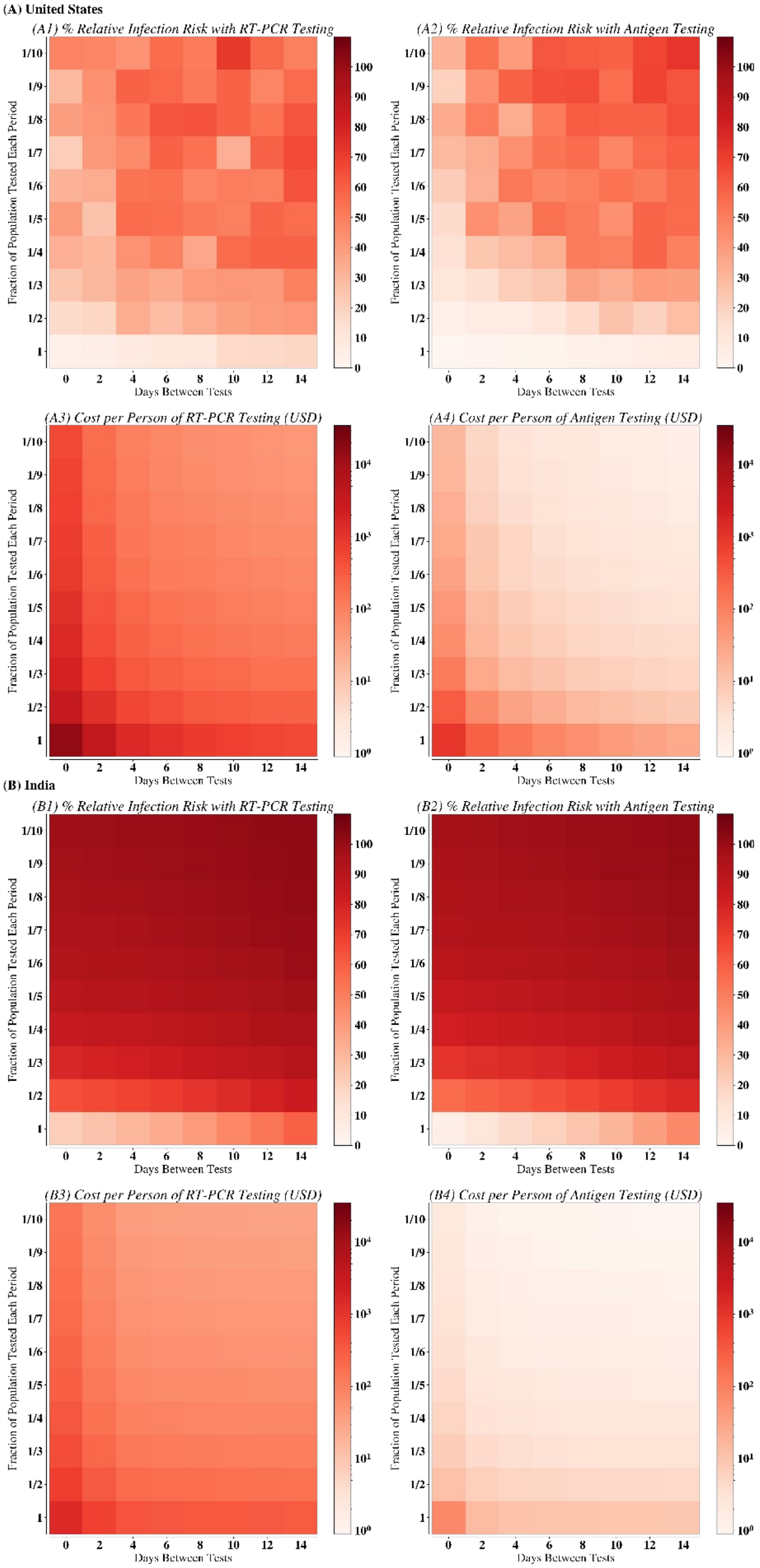

Figure S5: Lower bound on results shown in Fig. S3 and Figs. 1 and 2.

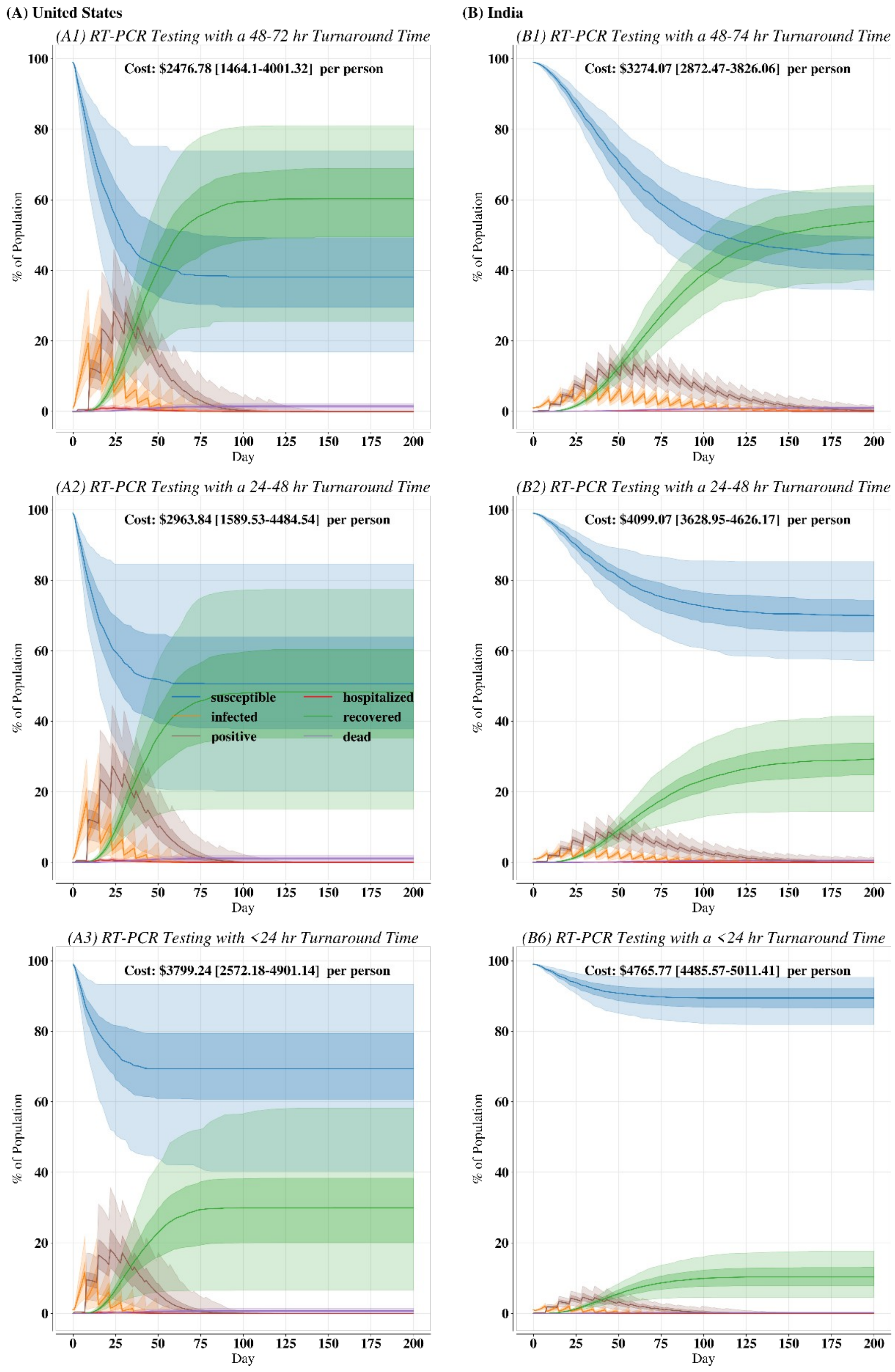

Figure S6: The impact of turnaround times in RT-PCR testing for (A) the US and (B) India when 100% of the population is RT-PCR tested weekly.

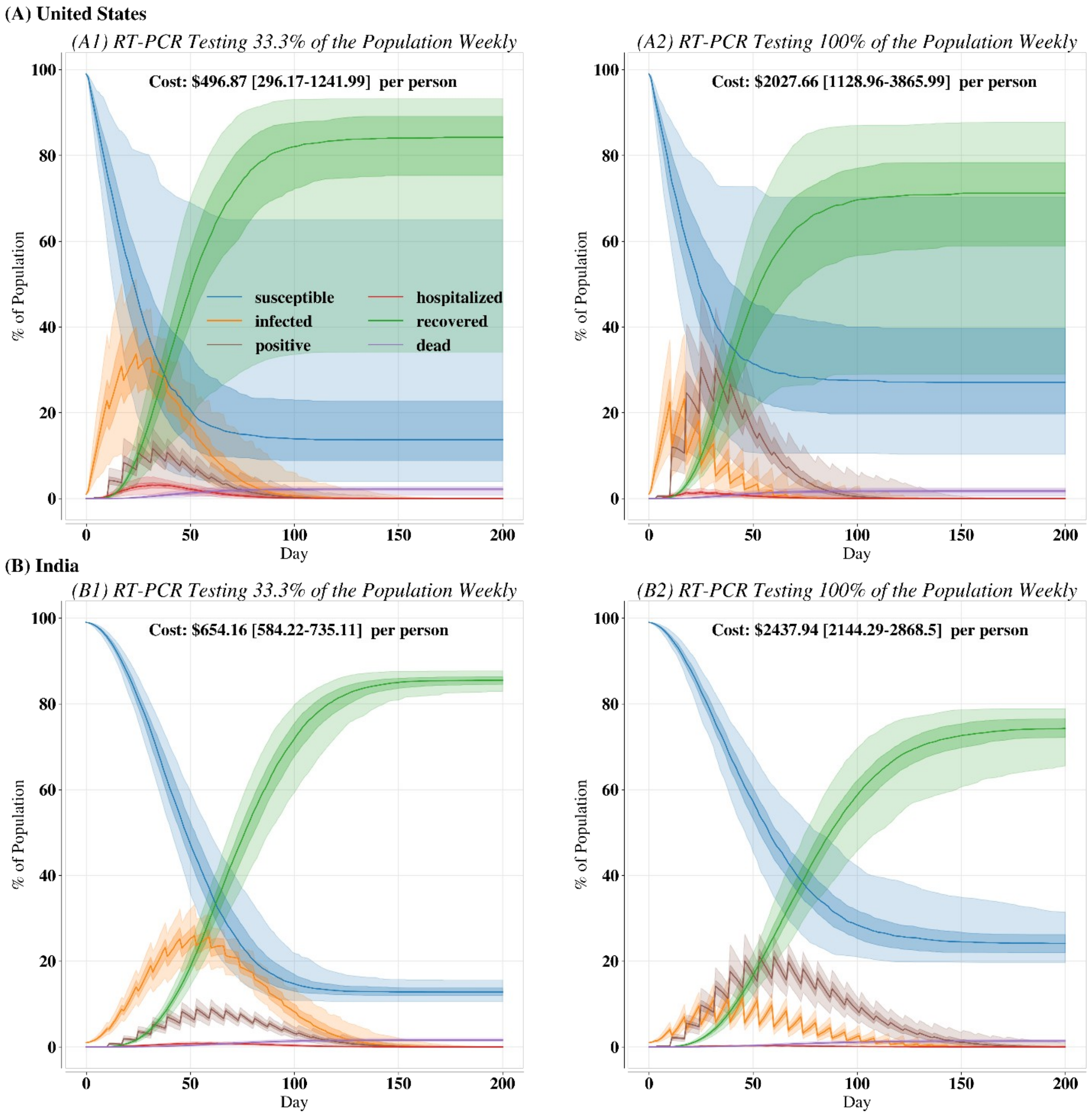

Figure S7: Exploratory RT-PCR testing scenarios for (A) United States and (B) India. (1) shows the disease course with 33.3% of the population tested one day once a week; (2) shows the disease course with 100% of the population tested one day once a week. Bold lines are the median over 200 independent replicates. Dark-shaded regions show 25th to 75th percentiles. Light-shaded regions are 2.5^{th} to 97.5^{th} percentiles. The cost estimates assume each individual without an infection is tested weekly. The sawtooth profile occurs because testing happens on a certain day and thus infected individuals are labelled as positive, resulting in a sudden decrease in infected individuals and an increase in positive individuals.

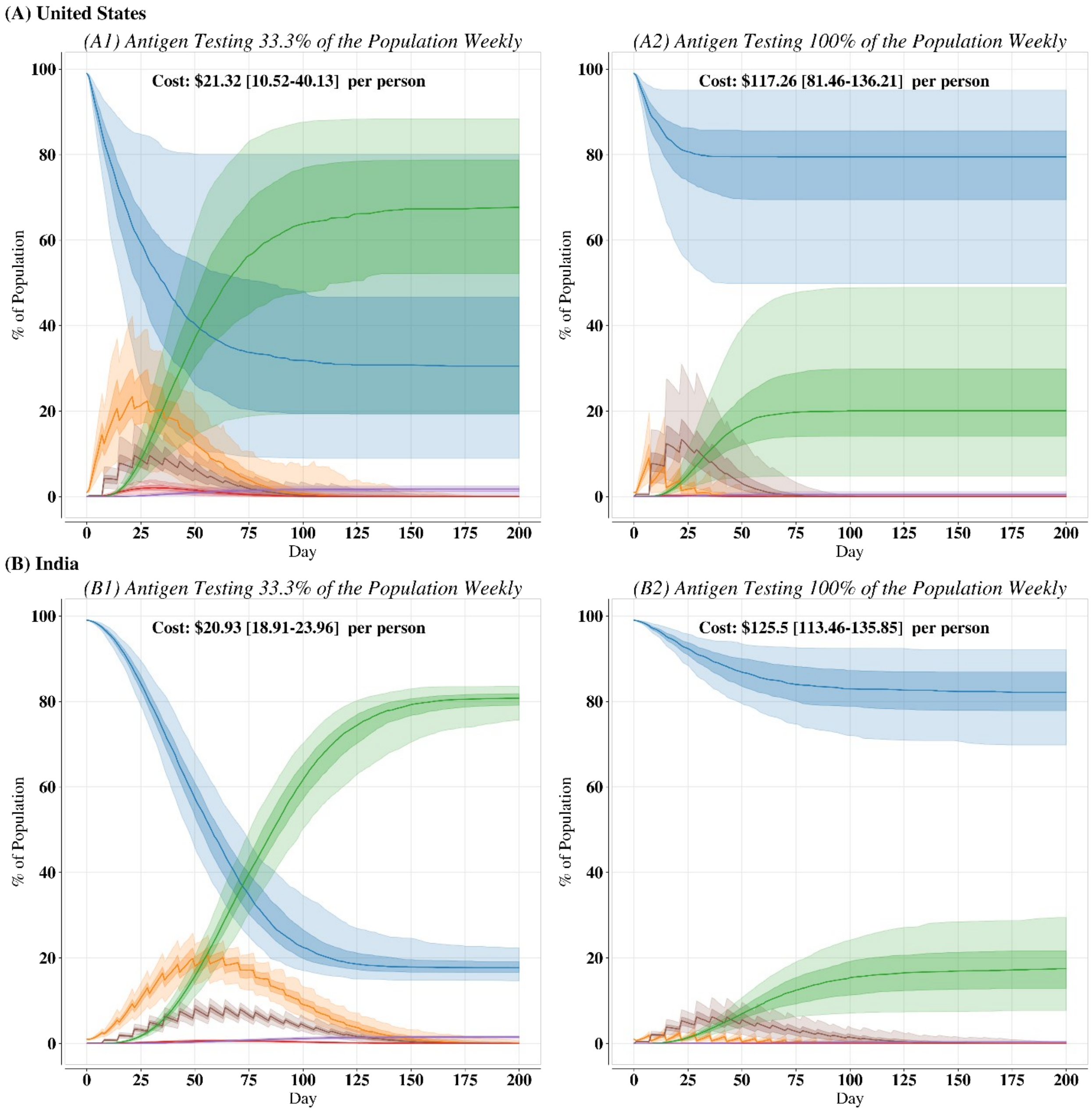

24

Figure S8: Exploratory antigen testing scenarios for (A) United States and (B) India. (1) shows the disease course with 33.3% of the population tested one day once a week; (2) shows the disease course with 100% of the population tested one day once a week. Bold lines are the median over 200 independent replicates. Dark-shaded regions show 25th to 75th percentiles. Light-shaded regions are 2.5th to 97.5th percentiles. The cost estimates assume each individual without an infection is tested weekly. The sawtooth profile occurs because testing happens on a certain day and thus infected individuals are labelled as positive, resulting in a sudden decrease in infected individuals and an increase in positive individuals.

25

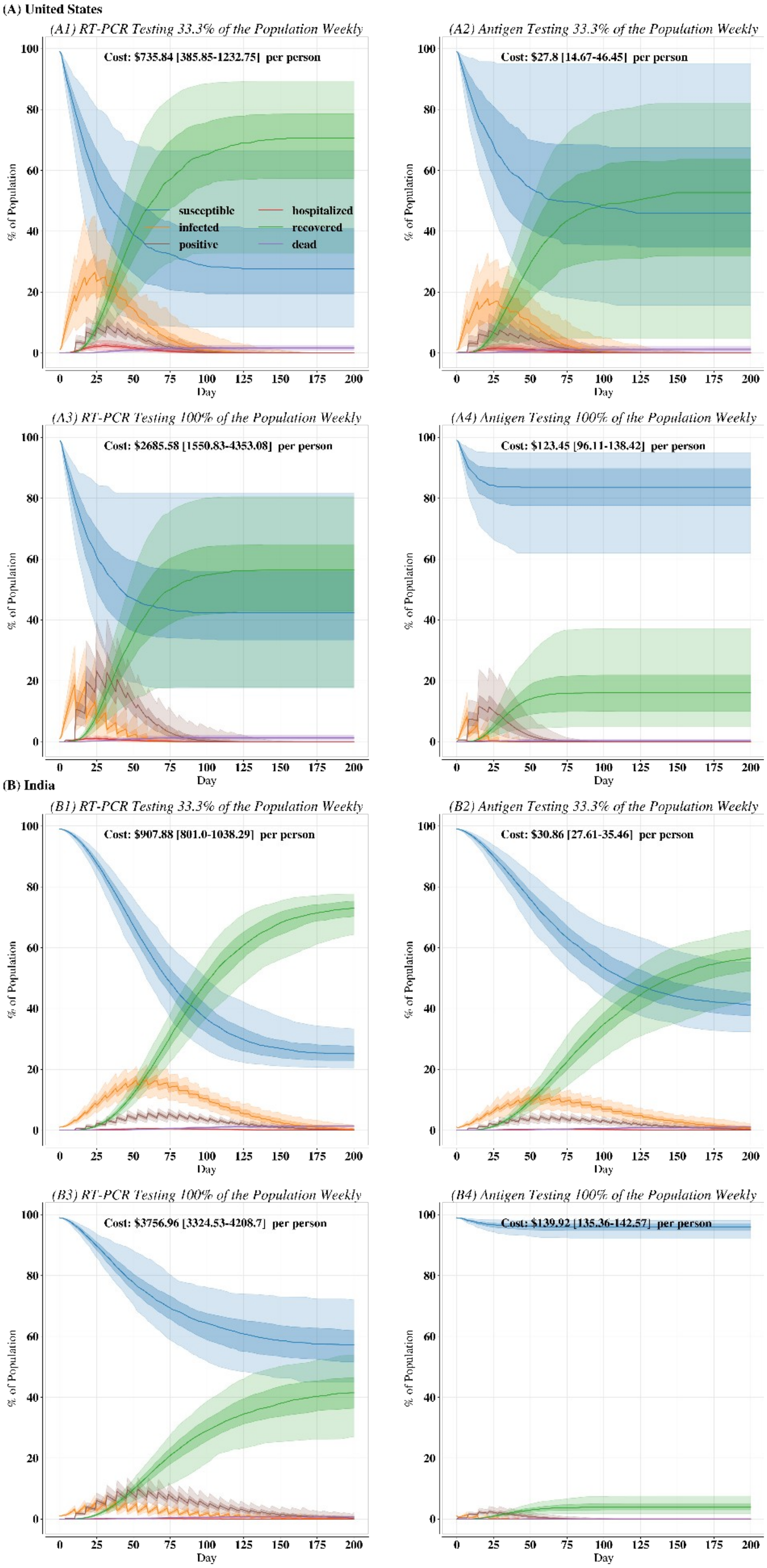

Figure S9: Exploratory testing scenarios in Fig. S7 with R_0 = 2.

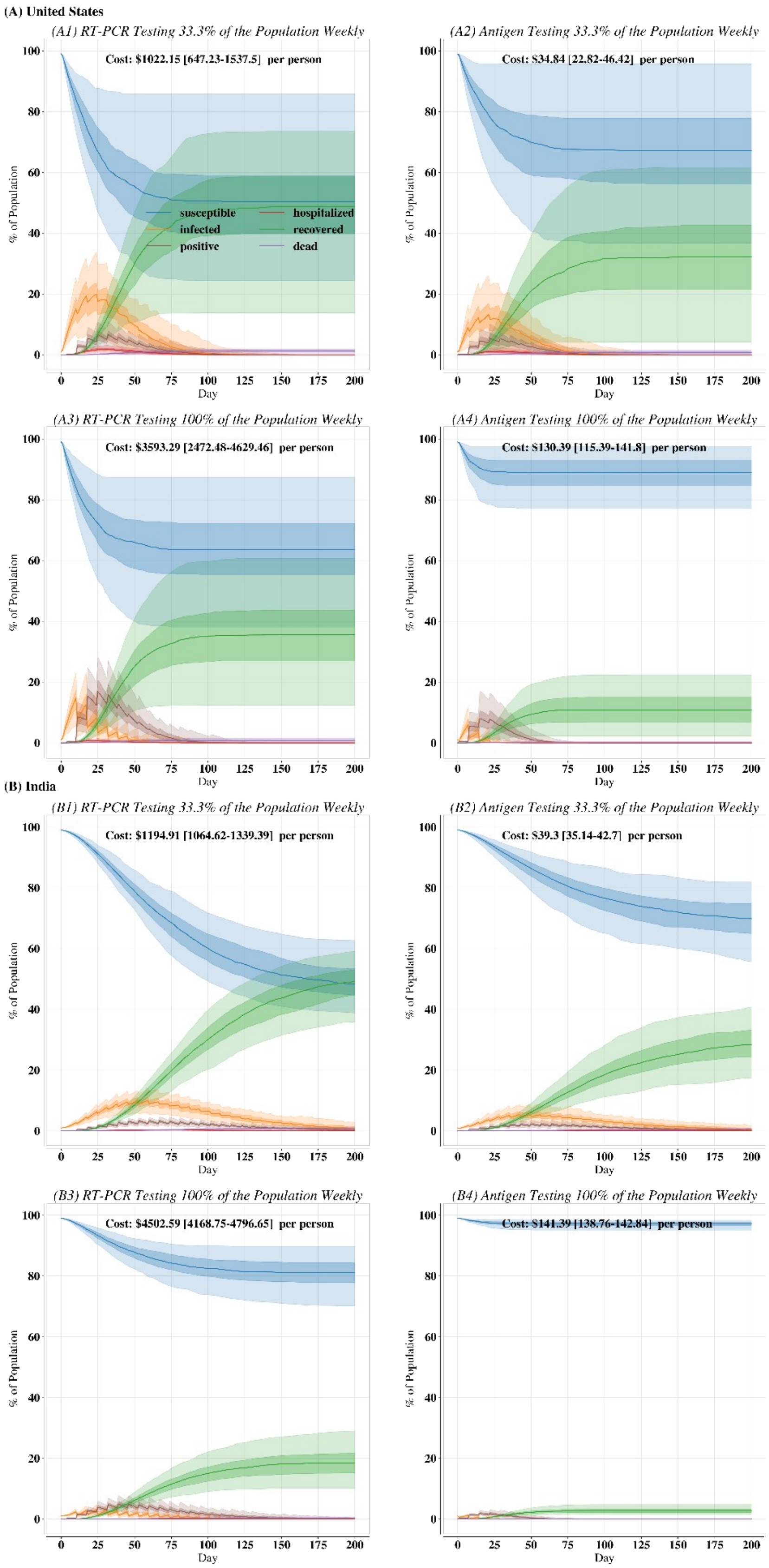

Figure S10: Exploratory testing scenarios in Fig. S7 with R_0 = 1.5.

Table S3: Standardized overall z-score regression results for predicting relative infection risk in each transmission setting by using testing at a certain coverage *g* (i.e., number of testing iterations required to surveil the full population), the frequency *f* (i.e., 1/(*d* + 1) where *d* is the number of days between testing occurrences), and the test used *r* (i.e., is the test used an RT-PCR assay?). The equation takes the form (A*g* + B*f*)/*gf* + C*r* where A, B, and C are the coefficients for *g*, *f*, and *r*, respectively. In this form, the numerator describes the risk of this testing procedure and the denominator standardizes the testing regiment by the characteristic time scale of the procedure; however, note that this equation can be arranged to be a linear function of 1/*f* and 1/*g*. Note that while Fig. 3 and 4 shows days between tests of 0, 4, 8, and 12 and fraction of population tested 1, ½, ¼, and 1/8, the regression equations are fit with 0, 2, 4, 6, 8, 10, 12, and 14 days between tests and 1, ½, 1/3, ¼, 1/5, 1/6, 1/7, 1/8, 1/9, and 1/10 as the fraction of the population tested.

	United States	India
Coefficient for *g*	0.2506 [0.2107 – 0.2906]	0.1913 [0.1591 – 0.2235]
Coefficient for *f*	-0.9300 [-0.9700 – -0.8901]	-0.9578 [-0.9900 – -0.9256]
Coefficient for *r*	0.1265 [0.0701 – 0.1830]	0.0930 [0.0475 – 0.1385]
R^2	93.5%	95.8%
F-statistic (p-val)	763.1 (2.33e-93)	1204. (4.44e-108)
AIC	20.6872	-48.42

Table S4: Same results as in S3 but now for only RT-PCR testing. The equation takes the form (A*g* + B*f*)/*gf* where A and B are the coefficients for *g* and *f*, respectively.

	United States	India
Coefficient for *g*	0.2744 [0.2349 – 0.3139]	0.1999 [0.1591 – 0.2408]
Coefficient for *f*	-0.9455 [-0.9850 – -0.9060]	-0.9629 [-1.0038– -0.9221]
R^2	96.9%	96.6%
F-statistic (p-val)	1232. (9.66e-60)	1149. (1.36e-58)
AIC	-47.7118	-42.2924

Table S5: Same results as in S3 but now for only antigen testing. The equation takes the form (A*g* + B*f*)/*gf* where A and B are the coefficients for *g* and *f*, respectively.

	United States	India
Coefficient for *g*	0.2402 [0.1918 – 0.2886]	0.1871 [0.1500 – 0.2243]
Coefficient for *f*	-0.9467 [-0.9951 – -0.8983]	-0.9684 [-1.0056– -0.9313]
R^2	95.3%	97.2%
F-statistic (p-val)	807.8 (7.37e-53)	1397. (8.27e-62)
AIC	-15.2036	-57.4782

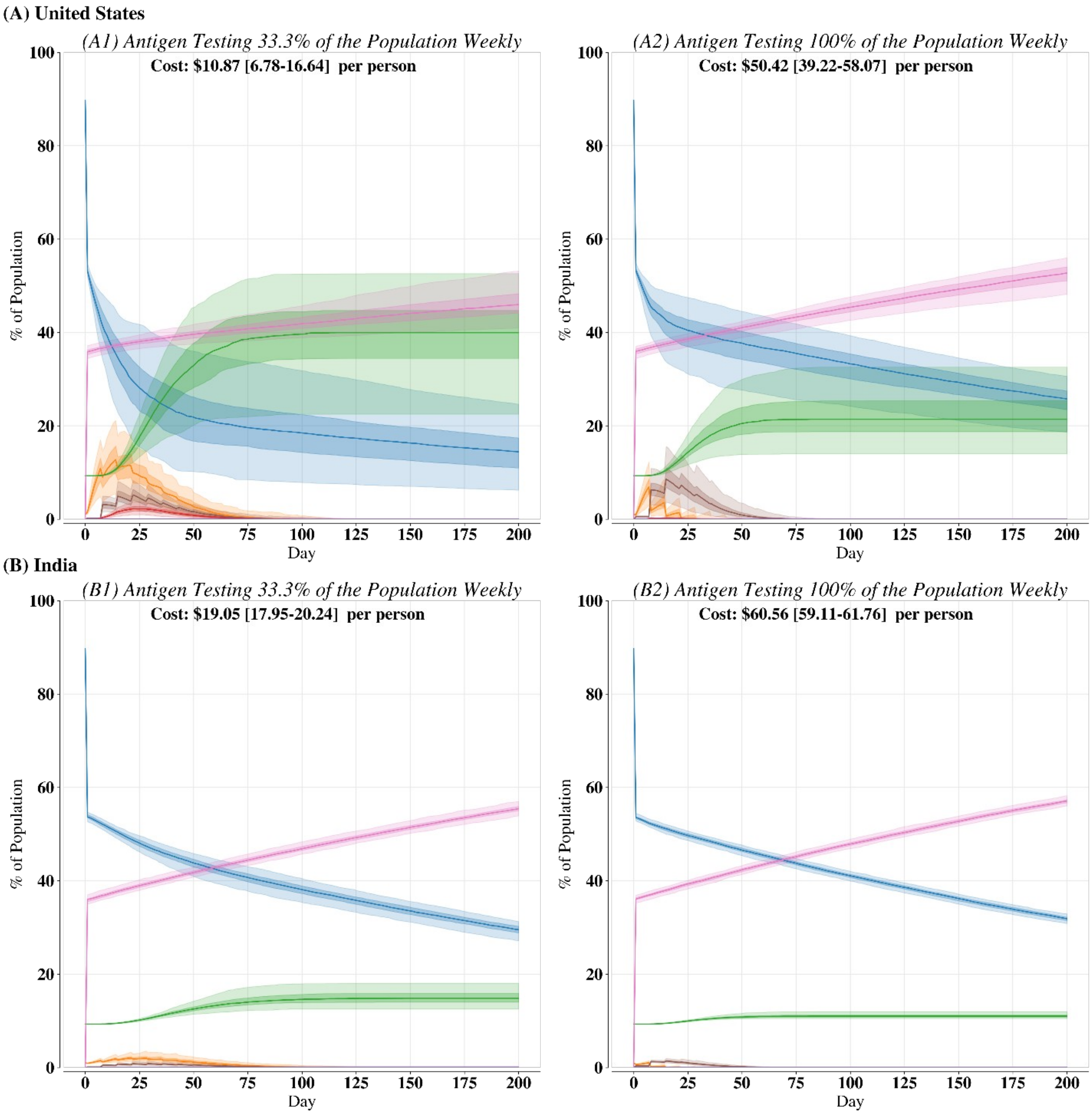

Figure S11: Disease course for (A) United States and (B) India with 40% of the population as initially vaccinated and then 0.25% of the susceptible population being vaccinated each subsequent day; vaccines are coupled with antigen testing (1) 33.3% or (2) 100% of the population weekly. Bold lines are the median over 200 independent replicates. Dark-shaded regions are the 25th to 75th percentiles. Light-shaded regions are 2.5th to 97.5th percentiles.

48

1. Abbott, S. K. and B. What Kind of Covid Test Should I Get? Answers on Cost, Accuracy and More. *Wall Street Journal* (2020).
2. Stites, E. C. & Wilen, C. B. The Interpretation of SARS-CoV-2 diagnostic tests. *Med* (2020).
3. He, X. *et al.* Temporal dynamics in viral shedding and transmissibility of COVID-19. *Nat. Med.* **26**, 672–675 (2020).
4. Kleiboeker, S. *et al.* SARS-CoV-2 Viral load Assessment in Respiratory Samples. *J. Clin. Virol.* 104439 (2020).
5. McAloon, C. *et al.* Incubation period of COVID-19: a rapid systematic review and meta-analysis of observational research. *BMJ Open* **10**, e039652 (2020).
6. Laxminarayan, R. *et al.* Epidemiology and transmission dynamics of COVID-19 in two Indian states. *Science* **370**, 691–697 (2020).
7. Hirotsu, Y. *et al.* Comparison of automated SARS-CoV-2 antigen test for COVID-19 infection with quantitative RT-PCR using 313 nasopharyngeal swabs, including from seven serially followed patients. *Int. J. Infect. Dis.* **99**, 397–402 (2020).
8. Hoertel, N. *et al.* A stochastic agent-based model of the SARS-CoV-2 epidemic in France. *Nat. Med.* **26**, 1417–1421 (2020).
9. Endo, A., Abbott, S., Kucharski, A. J. & Funk, S. Estimating the overdispersion in COVID-19 transmission using outbreak sizes outside China. *Wellcome Open Res.* **5**, 67 (2020).
10. Zhang, Z., Hou, Y., Li, D. & Li, F. Diagnostic efficacy of anti-SARS-CoV-2 IgG/IgM test for COVID-19: A meta-analysis. *J. Med. Virol.* (2020).
